# Quarterly Retrospect

**Published:** 1862-07

**Authors:** 


### *Quarterly Retrospect.*

*July*, 1862.

The past three months have been crowded with events of unusual interest. Of these, first in order of importance, if not of time, was the meeting of the National Association for the Promotion of Social Science, under the presidency of Lord Brougham, in the Metropolis. Although the meeting occurred at a time when London was in the full whirl of "the Season;" when Parliament and the Courts of Law were sitting; when every one was living at the highest tension of his life, whether that were of business or pleasure; the Association suffered less than might have been anticipated. Nay, more; notwithstanding the difficulties it had to contend with, it achieved a splendid success. The great evening reception of the Members, in the Palace of Westminster, was a unique event in the social history of the nation, and is of historical importance. Nothing could have more remarkably shown the sympathy with which the voluntary efforts of the Association for the weal of the people are regarded by those who are best capable of giving those efforts practical effect, than opening to its members, for their peculiar gratification, the magnificent halls occupied by the Legislature, and hitherto sacred to it alone.

This meeting of the Social Science Association was, moreover, of international importance, from its being held in conjunction with the *Congrès International de Bienfaisance*, which held its meetings in Burlington House, while the Social Science Association sat at the Guildhall.

If the meeting of the different departments of the latter Association were of necessity somewhat meagrely attended, this was compensated by the facts, that the most active workers were rarely absent, and that the numerous papers read and discussed, probably surpassed in interest and importance those presented at any previous meeting. The meeting would probably fail in exercising that effect on the public mind which it was to be desired that it should have had, from the daily press being prevented, in consequence of the space taken up by reports of the debates in Parliament, giving any but the most scanty notices of its proceedings. Any just criticism of the work done in the different departments is, from this cause, impracticable, until the Transactions of the Association for the present year are published.

The meeting was inaugurated by service in Westminster Abbey, when the Very Rev. the Dean of Chichester preached to the Members; and in the evening of the same day Lord Brougham delivered an address to the Association in Exeter Hall. This address was characterized by its great scope and rare vigour; the force of the sentences contrasting strangely, almost painfully, with the enfeebled and almost inaudible utterance of the deliverer. Nothing having an important bearing upon the interests and prospects of Social Science at home or abroad seemed to be overlooked. Certain of his Lordship's observations have a special interest for the readers of this journal. The remarks referred to are as follows:—

"The great evil—indeed, the extreme danger of occasional legislation, has often been acknowledged. Some abuse gives rise to complaint; the misconduct of a public functionary has had mischievous effects; or the proceedings of a Court have given general dissatisfaction, and a remedy is desired. But it may be that the measure adopted to prevent a recurrence of the evil is merely such as the mischief complained of has suggested, and is devised without due regard to other circumstances; it may be one-sided and confined to the single case that has caused complaint; it may go too far, and, on account of an accidental miscarriage, make a general change in the law; or it may fall short of the improvement which sound views require, by being empirically confined to the peculiar circumstances of the case. Nothing can be more fit than that the occurrence of actual mischief showing the defects in the existing law should occasion a legislative change; but the greatest care should be taken to frame any proposed alteration upon enlarged views, and not confine it to suit the particular case. A late trial of lunacy gave rise to great and general complaint of the judicature, from the prolixity of the proceedings, and the irrelevancy of many matters introduced—their irrelevancy to the one question before the Court. A Bill has been introduced to improve the procedure in lunacy generally, and there is great reason to apprehend that it seeks, in material respects, unnecessarily to alter the law. One important and very salutary change is the requiring each cause to be tried by a judge instead of an officer of the Court, whose authority with counsel cannot be such as duly to control their proceedings. But a most unwise, and, indeed, absurd restriction is imposed upon the admission of evidence, which would have been wrong under the old procedure, but is wholly unintelligible now that a judge is to preside. That the late trial showed the necessity of some change is admitted; but the objection is, that the change goes beyond the exigency of the case, and in a wrong direction. It is plainly made to meet the outcry against portions of the evidence adduced.

On the motion of the Earl of Derby (May 9th) a Committee has been appointed by the House of Lords to inquire into the injurious effects of noxious vapours in certain manufacturing processes. From the illustrations brought forward by his Lordship in support of the motion, it is most probable that many curious and interesting facts will be brought to light of the influence of such vapours upon animal and vegetable life.

It would almost seem as if the horrible and brutalizing events of the civil war in America had infected the population of this kingdom with a thirst for blood. Not only has agrarian murder—the most abominable and revolting of all kinds of murder—become again prevalent in several districts of Ireland, and brought about once more the sad necessity of issuing Special Commissions, but in England also murder has broken out almost as an epidemic, and is of a less foul character than the outbreak in Ireland, only because not the birth of a deliberate system fostered by certain sections of the population. Even now as we write, with our notes heaped up with horrid details, jotted down since our last Retrospect, the day's paper brings to us the news of still another most barbarous and revolting murder of a young lady in one of the southern counties—a murder perpetrated in the open fields, and preceded by the vilest lust.

But murder, the result of unrestrained passion, is that form of all others which we can least hope to diminish in amount or frequency. So long as there are men to be found, who in their ignorance and habits are but brute beasts in human form, so long will the restraints of the law be broken through. But there is one class of murders, and that of the most hideous description, indeed, very commonly multiple, the frequent occurrence of which is a disgrace to a civilized country. We allude to MURDERS BY UNRECOGNISED LUNATICS. Again we must darken our pages with illustration after illustration of murders of this class. Scarcely an Assize now passes but one or more examples of murder by lunatics whose lunacy had not been recognised, although *characterized by conspicuous signs*, is brought under the consideration of the bench and jury, who of necessity acquit the man. But it would seem as if neither judges, nor juries, nor the press, nor the public, would deal with this common-place fact in a common-sense manner. The moment the question of insanity is broached, almost every voice is raised in outcry against the plea, as if the plea were a simple medical quibble, legally used to save a man from the gallows. They will not see that the saving of a supposed or manifest lunatic from a malefactor's death is the least part of the grave question at issue. To hang an insane man is illegal, and it is every man's duty, little though he may think it, to prevent such an illegality. Decency, moreover, as well as law, requires that incoherent lunatics, such as George Clark,* should not be sentenced to death as eccentrics one day and respited as lunatics the next. In acknowledging the simple, unquestionable fact of many murders being committed by unrecognised lunatics, it should also be acknowledged that many of these murders are capable of prevention. This is the great doctrine with which Alienist physicians have hitherto in vain sought to inoculate the people and the press. There is a horrible sameness in murders of this

---

* Tried for murder at Newcastle, 29th February last. The case is reported in our last number, p. 304.

class. As in the latest instance, the murder of two children by their father, and suicide of the murderer, in Blackfriars-road (June 11th); also in the murder of two children on Ludgate-hill by the mother, and attempted suicide of the murderess (7th June); also in the threatened murder and deliberate suicide at Birmingham (8th June)—we find in each case accounts of preceding unheeded mental disturbance in the murderer—that is to say, a marked and conspicuous change from previous habits and dispositions, with gloomy thoughts and tendencies. Had a like change taken place in the function of the stomach or the heart, of an arm or a leg, the doctor would have been hastily sought: but the mental change arising from disease is suffered to go on day after day, and treated as a trifling thing, or put down to "temper," or some present annoyance, until it culminates in a delusion or impulse of which murder of those most cherished is a *natural* result. Let the public learn to look upon lunacy as a disease to which they are as much exposed as to maladies of the lungs, of the heart, or the bowels: let them learn to apply to a physician when they observe strange alterations in the ordinary mental functions of an individual, as readily as they seek his help for changes experienced in the bodily functions; and we shall presently see a diminution of those sad, those horrible murders which are the direct results of a *diseased mind*—of lunacy. Let the public, in short, learn to recognise lunacy as they recognise bodily disease. This is the only true doctrine of "fact" in lunacy.

### THE CASE OF GEORGE CLARK.

An anti-climax has to be recorded in the case of George Clark, sentenced to death at Newcastle for the murder of Mark Frater, but respited on account of lunacy, and commented upon in the last number of the Journal. The magistrates having persisted in their refusal to sign the necessary certificates for the removal of the subsequently proved lunatic to an asylum, the Home Secretary has found it necessary to commute the sentence upon the murderer to transportation for life. In this manner the necessity of hanging a lunatic has been at length avoided. The magistrates refused to confirm the certificates of lunacy for the reason that they considered the grounds upon which the medical opinions were based insufficient to constitute lunacy.

